# Renal angina index predicts fluid overload in critically ill children: an observational cohort study

**DOI:** 10.1186/s12882-021-02540-6

**Published:** 2021-10-11

**Authors:** Stephen M. Gorga, Erin F. Carlton, Joseph G. Kohne, Ryan P. Barbaro, Rajit K. Basu

**Affiliations:** 1 Department of Pediatrics, University of Michigan Medical School, 1500 E. Medical Center Drive, F-6890, Ann Arbor, MI 48109 USA; 2 Susan B. Meister Child Health Evaluation and Research (CHEAR) Center, University of Michigan, Ann Arbor, MI USA; 3 Children’s Healthcare of Atlanta/Emory University, Atlanta, GA USA

**Keywords:** Fluid overload, Acute kidney injury, Renal angina index, Kidney disease: improving global outcomes

## Abstract

**Background:**

Fluid overload and acute kidney injury are common and associated with poor outcomes among critically ill children. The prodrome of renal angina stratifies patients by risk for severe acute kidney injury, but the predictive discrimination for fluid overload is unknown.

**Methods:**

Post-hoc analysis of patients admitted to a tertiary care pediatric intensive care unit (PICU). The primary outcome was the performance of renal angina fulfillment on day of ICU admission to predict fluid overload ≥15% on Day 3.

**Results:**

77/139 children (55%) fulfilled renal angina (RA+). After adjusting for covariates, RA+ was associated with increased odds of fluid overload on Day 3 (adjusted odds ratio (aOR) 5.1, 95% CI 1.23–21.2, *p* = 0.025, versus RA-). RA- resulted in a 90% negative predictive value for fluid overload on Day 3. Median fluid overload was significantly higher in RA+ patients with severe acute kidney injury compared to RA+ patients without severe acute kidney injury (% fluid overload on Day 3: 8.8% vs. 0.73%, *p* = 0.002).

**Conclusion:**

Among critically ill children, fulfillment of renal angina was associated with increased odds of fluid overload versus the absence of renal angina and a higher fluid overload among patients who developed acute kidney injury. Renal angina directed risk classification may identify patients at highest risk for fluid accumulation. Expanded study in larger populations is warranted.

**Supplementary Information:**

The online version contains supplementary material available at 10.1186/s12882-021-02540-6.

## Background

Fluid overload (FO) and acute kidney injury (AKI) are common and both are associated with poor outcomes among critically ill children [1–5]. Over 25% of all children admitted to a pediatric intensive care unit (PICU) experience AKI and severe AKI is independently associated with a 12% mortality rate [5]. Similarly, FO occurs in over one-third of critically ill children and it is consistently associated with increased mortality, mechanical ventilation duration, hospital and intensive care unit (ICU) length of stay [1, 2]. Furthermore, emerging evidence suggests concurrent FO and AKI worsens outcomes significantly [6].

**Fig. 1:**
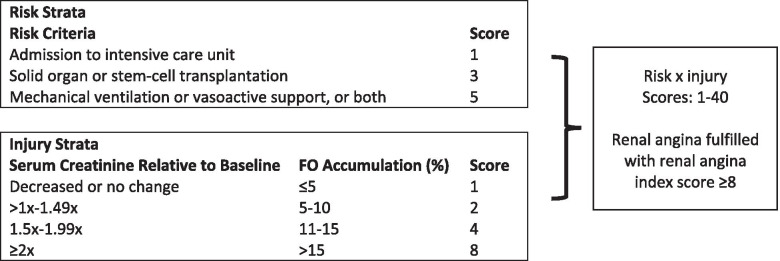
The renal angina index. Adapted from Basu, et al. [10]. Renal angina index is calculated by multiplying the Risk Strata score by the Injury Strata score. The score assigned in the Injury Strata is the highest score based on either the serum creatinine or FO accumulation. The score is calculated at 12 h after ICU admission. FO: Fluid overload, by percentage

There is no definitive therapy for AKI after it occurs [7]. In the absence of proven restorative therapy, prevention of AKI has been identified as a priority for management of at risk critically ill patients [7]. Identification of these patients appears to be possible using the renal angina (RA) prodrome for AKI risk stratification [8, 9]. The Renal Angina Index (RAI) is a validated measurement of RA that combines patient-specific risk factors as well as early signs of renal dysfunction [9, 10]. (Figure 1) Scored at 12 h into a hospitalization course, the RAI can predict the development of severe AKI on Day 3 of hospitalization with a high negative predictive value (NPV) [9–12]. Utilizing the RAI early in a hospitalization to identify patients at risk for severe AKI provides an opportunity for the development of targeted, patient-centered care to prevent AKI.

Similar to AKI, FO after Day 3 of hospitalization is associated with poor outcomes [13–16]. Further, higher peak FO and the rate of accumulation of FO is associated with considerable mortality and morbidity [1, 2]. Randomized and observational trial data demonstrate consistent benefit of targeting neutral fluid balances in the ICU and clinicians report management aligned with these evidence-based practices [17–21]. Despite this, evidence suggests that in daily practice, clinicians fail to recognize nearly one-third of patients who have significant FO and patients continue to receive unrecognized levels of fluid administration [19, 22, 23]. This delayed recognition likely contributes to worse outcomes [14, 19]. Currently, there are no instruments to assist clinicians’ identification of patients at risk for the development of significant FO.

While the RAI can identify patients who are at high risk for AKI, its ability to identify patients who are at risk for FO is unknown. We therefore investigated the predictive efficacy of the RAI to identify significant FO among critically ill children after the initial resuscitative period. We hypothesize that RA would be associated with higher FO states on Day 3 after ICU admission.

## Methods

### Study population

A prospective, observational convenience cohort of patients admitted to a PICU at a tertiary care children’s hospital from March 2017 through August 2018 was evaluated, as part of a larger study of clinician identification and prediction of multiple organ dysfunction syndrome in children [24]. All patients included in the parent study period had an expected length of stay greater than 2 days at admission determined by a member of the care team as previously described. For our current study, patients were required to have a serum creatinine measurement within 12 h of ICU admission as well as complete fluid balance data through Day 3 after ICU admission. Patients were also required to have the complete data necessary to determine RAI, including transplant, mechanical ventilation, and vasoactive medication status. Exclusion criteria were death before Day 3, history of stage 5 chronic kidney disease (i.e., estimated glomerular filtration rate [eGFR] < 15 mL/min per 1.73m^2^ or on maintenance dialysis), kidney transplantation in the last 90 days, incomplete kidney injury information, or patients admitted during the study period but were not part of the parent study. All methods were carried out in accordance with relevant ethics guidelines and regulations and approved by the University of Michigan. The Institutional Review Board (IRB) at the University of Michigan approved this study and because standard of care was not altered in any way, written informed consent was waived for patients.

### Data collection

Patients’ medical records were reviewed for creatinine measurements, past medical history, total fluid input and total fluid output. Hourly urine output was recorded by PICU nursing staff per unit protocol. In an effort to minimize false positive identification of oliguria, in patients without urethral catheters, the total output of mixed stool and urine was used to define urine output as previously published [25]. Additionally, if the patient had an unmeasurable output, it was defined as a normal amount for age by default. To further protect against false positive identification of oliguria in patients without urethral catheters, total urine output was divided over the period of time between instances of output, allowing for normal sleep periods of > 6 h with no urine output. This protected false identification of stage 1 AKI by Kidney Diseases: Improving Global Outcomes (KDIGO) [26].

### Definitions

Baseline creatinine was measured based on the lowest measured creatinine in the previous 3 months prior to hospitalization [27–29]. If the patient was hospitalized for the last 3 months prior to PICU admission, the lowest creatinine measurement more than 1 month prior to PICU admission was used. If this measurement was not available, baseline creatinine was imputed using age dependent calculations with the assumption of a glomerular filtration rate (GFR) of 120 ml/min/1.73m^2^, as previously published [29]. We utilized KDIGO staging criteria to define and classify AKI [26]. The worst stage achieved by serum creatinine or urine output was used to classify kidney injury stage. Severe acute kidney injury was defined as stage 2 or worse.

The renal angina index was determined after 12 h on the day of ICU admission as previously described, combining the risk and injury scores [9]. (Figure 1) A RAI score of 8 or more is considered fulfilment of renal angina (RA+) based on derivation and validation studies [9, 10].

Hourly FO was determined using intake and output. Because percent FO is part of the definition of RAI, cumulative percent FO started at 12 h after ICU admission, after RAI fulfilment. All subjects were normalized to 0% FO after RAI calculation and FO was calculated thereafter. Cumulative FO was then calculated every 12 h through 96 h according to previously published definitions [14]:

$$\mathrm{Percent}\ \mathrm{FO}=\frac{\mathrm{Sum}\ \mathrm{of}\ \mathrm{daily}\ \left({\mathrm{Fluid}\ \mathrm{Liters}}_{\mathrm{in}}-{\mathrm{Fluid}\ \mathrm{Liters}}_{\mathrm{out}}\right)\ \mathrm{x}\ 100}{\mathrm{ICU}\ \mathrm{admission}\ \mathrm{weight}\ \left(\mathrm{kg}\right)}$$

### Outcomes

The primary outcome was the amount of FO at Day 3 after ICU admission. Due to anticipated nonparametric data, FO as an outcome for regression models was dichotomized at 15% in order to harmonize multiple previously published definitions, accepted published FO data, and recent consensus statements regarding FO in various critically ill populations of children [1, 14, 30]. Secondary outcomes included differences in FO at 12 h increments through Day 3, as this is seen as clinically relevant time points for assessment and intervention after the period of resuscitation [7].

Furthermore, assessment of the predictive characteristics of RAI for severe AKI were explored. Given that we investigated the predictive characteristics of RAI for both AKI and FO, and these states can exist together or separately, patients were further characterized into 4 discrete phenotypes at Day 3 based on the presence or absence of FO and AKI: 1) FO+/AKI+, 2) FO−/AKI+,3) FO+/AKI-, and 4) FO−/AKI- [6]. Finally, we also investigated included the association of FO with ICU length of stay, hospital length of stay, renal replacement therapy utilization, and mortality at 30 days.

### Statistical analysis

Statistical analysis was performed using Stata 16 (StataCorp, LLC, College Station, TX, USA). Categorical data are presented as number and percentages and compared by χ^2^ or Fisher’s exact test. Continuous data are presented as medians and interquartile ranges (IQR), as we anticipated non-normally distributed data, and compared using the Mann-Whitney test. Repeated admissions were included and considered as a separate, discrete risk of developing AKI. We used bivariate and multivariate logistic models to correct for significant covariate effects and identify independent associations with outcomes. All bivariate associations carrying associations with *p* < 0.15 were included as multivariate model terms. A *p* value ≤0.05 was considered significant.

## Results

### Fluid overload

**Fig. 2:**
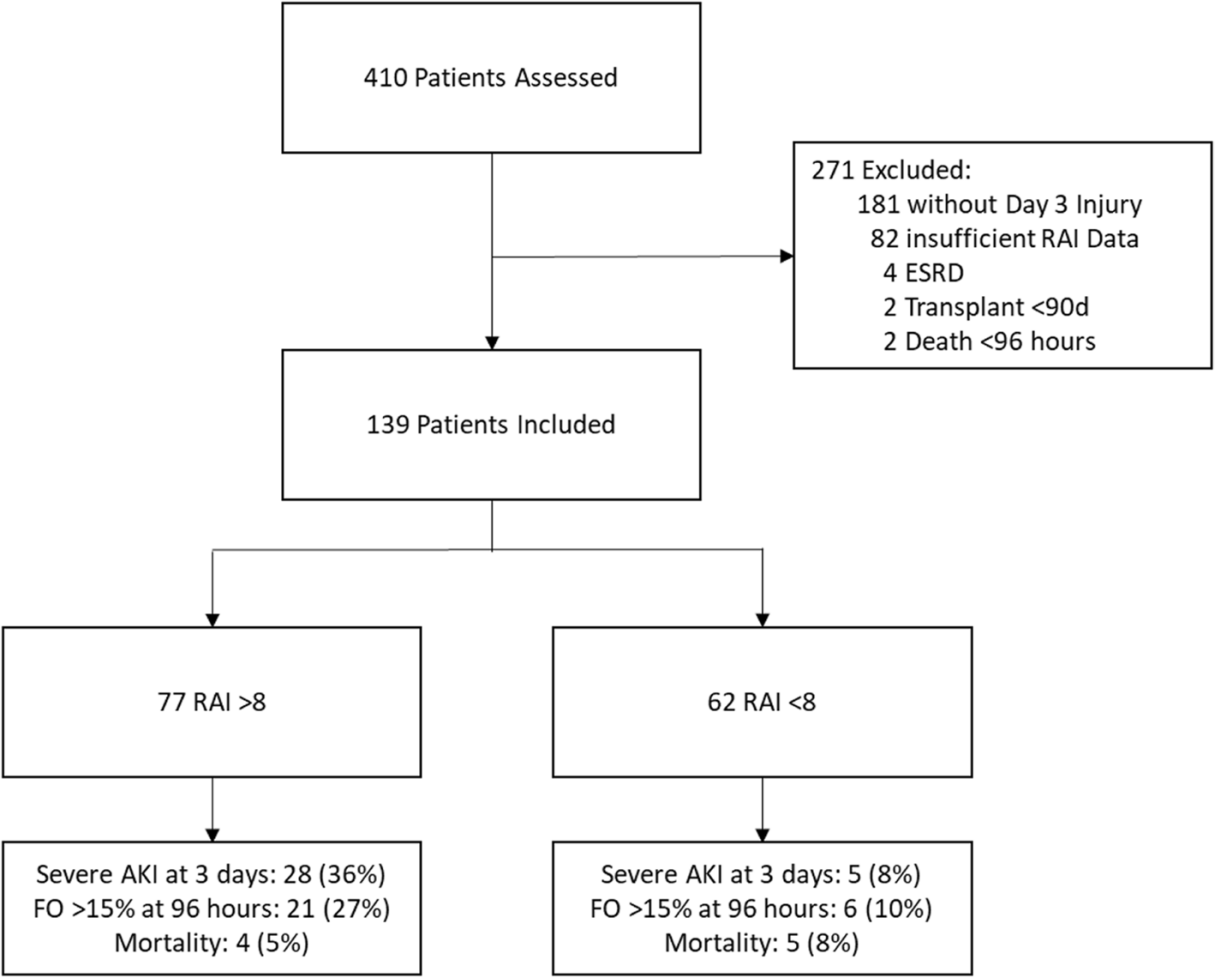
Participant Inclusion. Data are n(%). AKI: Acute kidney injury; ESRD: End Stage Renal Disease; RAI: Renal angina index; FO: Fluid overload

Of 410 children in the initial data collection, 139 (33.9%) met criteria for inclusion (Fig. 2). Seventy-seven of 139 (55%) children fulfilled RA criteria (RA+) (Table 1). Compared to the absence of RA (RA-), a higher proportion of RA+ had FO on Day 3 (27% vs. 10%, *p* = 0.009). This finding remained significant after correcting for severity of illness, age, mechanical ventilator status, transplant status, CRRT receipt, and presence of severe AKI (adjusted odds ratio (aOR) 5.1, 95% CI 1.23–21.2, *p* = 0.025). FO developed in 27/139 (19.4%) (Table 2). The negative predictive value of RA+ for FO Day 3 was 90.3% (80.1–96.4%) with an area under the ROC curve (AUROC) of 0.64 (0.55–0.73) (Supplemental Table 1).

**Table 1:** Renal angina fulfillment, patient demographics, and outcomes

Demographics	All Patients	RA-	RA+	***P***-value
	***N*** = 139	***N*** = 62	***N*** = 77	
Male	68 (48.9%)	35 (56%)	33 (43%)	0.11
Age (y)	6 (1.33–12.33)	6 (1.17–13)	6 (1.4–11)	0.57
PRISM III	6 (3–11)	3 (1–7)	9 (4–13)	< 0.001
Mechanical Vent	73 (52.5%)	20 (32%)	53 (69%)	< 0.001
CRRT	11 (7.9%)	1 (2%)	10 (13%)	0.014
Transplant	9 (6.5%)	0 (0%)	9 (12%)	0.005
Day 3 AKI Status				
None	88 (63.3%)	50 (81%)	38 (49%)	< 0.001
Stage 1	18 (12.9%)	7 (11%)	11 (14%)	0.60
Stage 2	13 (9.4%)	2 (3%)	11 (14%)	0.026
Stage 3	20 (14.4%)	3 (5%)	17 (22%)	0.004
Any Stage AKI	51 (36.7%)	12 (19%)	39 (51%)	< 0.001
Severe AKI	33 (23.7%)	5 (8%)	28 (36%)	< 0.001
Day 3 FO ≥15%	27 (19.4%)	6 (10%)	21 (27%)	0.009
ICU LOS	6 (3–11)	5.5 (3–10)	6 (3–12)	0.92
Hospital LOS	12 (7–23)	11 (6–21)	15.5 (8–31.5)	0.19
Mortality	9 (6.5%)	5 (8%)	4 (5%)	0.49

Data are presented as median (IQR) for continuous measures, and n (%) for categorical measures

*RAI*- Renal Angina Index negative, *RAI+* Renal angina index positive, *PRISM III* Pediatric risk of mortality 3, *CRRT* Continuous renal replacement therapy, *AKI* Acute kidney injury, stages defined by Kidney Disease: Improving Global Outcomes (KDIGO) criteria, *ICU* Intensive care unit. LOS: Length of stay

**Table 2:** Fluid overloaded ≥15% at Day 3, patient demographic characteristics, and outcomes

Demographics	All Patients	FO < 15%	FO ≥ 15%	***P***-value
	***N*** = 139	***N*** = 112	***N*** = 27	
Male	68 (48.9%)	57 (50.9%)	11 (40.7%)	0.34
Age (y)	6 (1.33–12.33)	6.745 (2–13)	1.4 (0.42–7)	0.002
PRISM III	6 (3–11)	5 (2.5–10)	9 (3–16)	0.077
Mechanical Vent	73 (52.5%)	60 (53.6%)	13 (48.1%)	0.61
CRRT	11 (7.9%)	1 (0.9%)	10 (37.0%)	< 0.001
Transplant	9 (6.5%)	6 (5.4%)	3 (11.1%)	0.28
Day 3 AKI Status				
None	88 (63.3%)	75 (67.0%)	13 (48.1%)	0.069
Stage 1	18 (12.9%)	16 (14.3%)	2 (7.4%)	0.34
Stage 2	13 (9.4%)	11 (9.8%)	2 (7.4%)	0.70
Stage 3	20 (14.4%)	10 (8.9%)	10 (37.0%)	< 0.001
Any Stage AKI	51 (36.7%)	37 (33.0%)	14 (51.9%)	0.069
Severe AKI	33 (23.7%)	21 (18.8%)	12 (44.4%)	0.005
RA+	77 (55.3%)	56 (50.0%)	21 (77.8%)	0.009
ICU LOS	6 (3–11)	6 (3–10)	6 (3–19)	0.28
Hospital LOS	12 (7–23)	11 (6–20)	29 (10–67)	0.003
Mortality	9 (6.5%)	7 (6.3%)	2 (7.4%)	0.83

Data are presented as median (IQR) for continuous measures, and n (%) for categorical measures

*RA-* Renal Angina Index negative, *RA+* Renal angina index positive, *PRISM III* Pediatric risk of mortality 3, *CRRT* Continuous renal replacement therapy, *AKI* Acute kidney injury, stages defined by Kidney Disease: Improving Global Outcomes (KDIGO) criteria, *ICU* Intensive care unit. *LOS* Length of stay

### Acute kidney injury

Severe AKI developed in 33/139 (23.7%) children (Table 3). RA+ was associated with risk of both Day 3 severe AKI (relative risk (RR) 4.5; 95% CI 1.85–10.99, *p* = 0.001) and Day 3 all stage AKI (RR 2.6, 95% CI 1.50–4.55, p = 0.001). The negative predictive value RA+ to predict severe AKI at Day 3 was 91.9% (82.2–97.3%), with an AUROC of 0.69 (0.62–0.77) (Supplemental Table 1). RA+ was also associated with increased renal replacement therapy (RRT) use, but not with mortality, ICU, or hospital length of stay (Table 1).

**Table 3:** Severe Acute Kidney Injury at Day 3, patient demographics, characteristics, and outcomes

Demographics	All Patients	No Severe AKI	Severe AKI	***P***-value
	***N*** = 139	***N*** = 106	***N*** = 33	
Male	68 (48.9%)	51 (48.1%)	17 (51.5%)	0.73
Age (y)	6 (1.33–12.33)	6 (1.17–12.3)	6 (1.75–13)	0.67
PRISM III	6 (3–11)	5 (2–9)	12 (7–19)	< 0.001
Mechanical Vent	73 (52.5%)	50 (47.2%)	23 (69.7%)	0.024
CRRT	11 (7.9%)	1 (0.9%)	10 (30.3%)	< 0.001
Transplant	9 (6.5%)	5 (4.7%)	4 (12.1%)	0.13
RA+	77 (55.3%)	49 (46.2%)	28 (84.8%)	< 0.001
Day 3 FO ≥15%	27 (19.4%)	15 (14.2%)	12 (36.4%)	0.005
ICU LOS	6 (3–11)	5 (3–10)	8 (4–13)	0.13
Hospital LOS	12 (7–23)	11 (6–20)	22 (9–50.5)	0.008
Mortality	9 (6.5%)	5 (4.7%)	4 (12.1%)	0.13

Data are presented as median (IQR) for continuous measures, and n (%) for categorical measures

*RAI-* Renal Angina Index negative, *RAI+* Renal angina index positive, *PRISM III* Pediatric risk of mortality 3, *CRRT* Continuous renal replacement therapy, *AKI* Acute kidney injury, stages defined by Kidney Disease: Improving Global Outcomes (KDIGO) criteria, *ICU* Intensive care unit. LOS: Length of stay

### Cumulative fluid trajectories

**Fig. 3:**
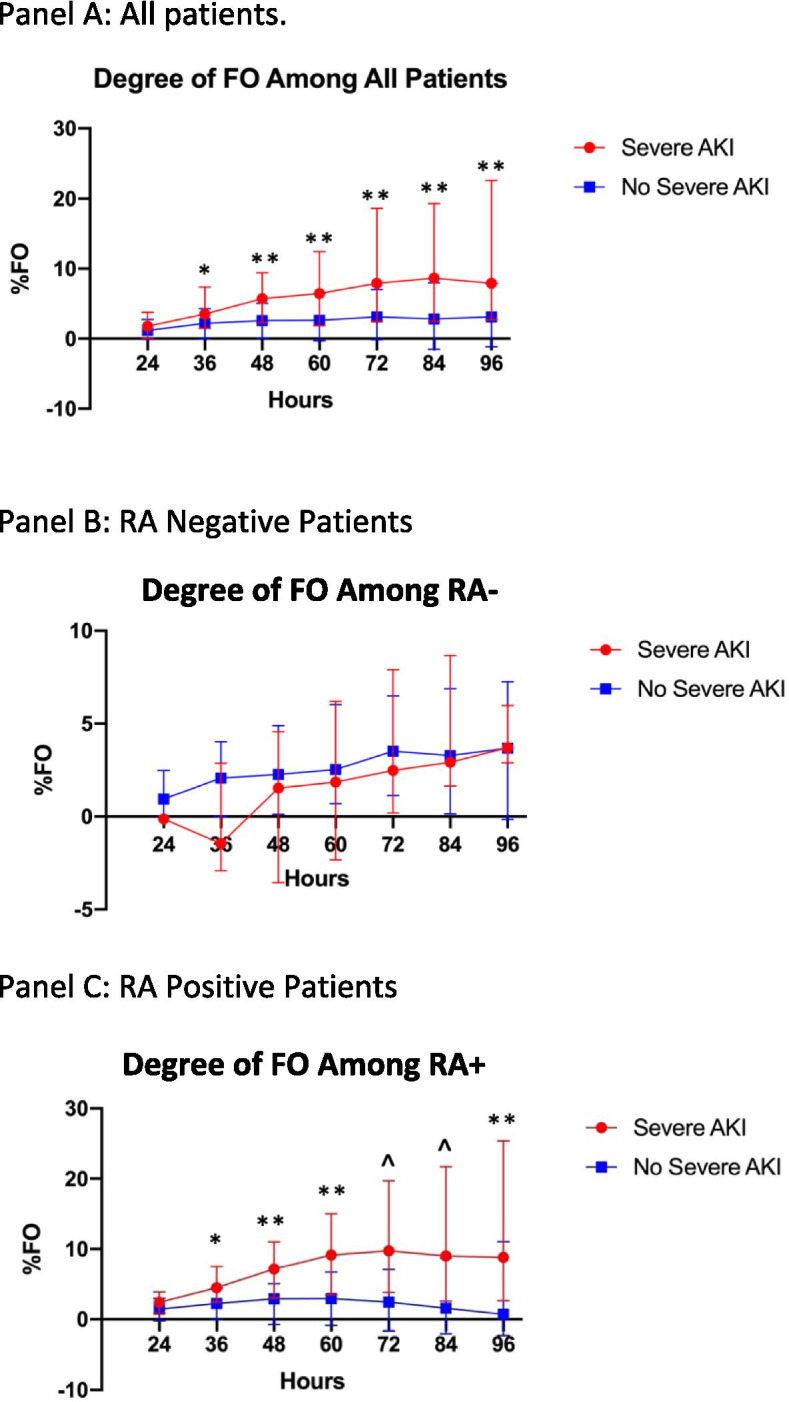
Cumulative FO percentage among patients over time since ICU admission. AKI: Acute Kidney Injury; FO: Fluid Overload: Hours: Time from ICU admission. *p < 0.05 **p < 0.005 ^*p* < 0.001.

In order to evaluate fluid trajectories among patients at risk for severe AKI by RA+, median FO% between patients who developed AKI were compared to those who did not. Median values were compared every 12 h from RAI scoring (Fig. 3) (Supplemental Table 2). Among RA+ patients, 28/77 (36%) developed severe AKI on Day 3. Median FO% was significantly higher among those who developed severe AKI on Day 3 compared to those who did not at each timepoint starting at 36 h (4.5% vs. 2.3%, *p* = 0.022) through 96 h (8.8% vs. 0.73%, *p* = 0.002) (Fig. 3A) (Supplemental Table 3). Among RA- patients, 5 of 62 (8%) developed severe AKI at Day 3. Fluid trajectories were not statistically different at any time point through 96 h between the two groups (3.7% severe AKI vs. 3.7% no severe AKI, *p* = 0.77) (Fig. 3B) (Supplemental Table 4). Among RA+ patients, FO at 96 h was independently associated with CRRT use and hospital length of stay, but not ICU length of stay or mortality at 30 days (Supplemental Table 5).

### Fluid overload and acute kidney injury phenotypes

**Fig. 4:**
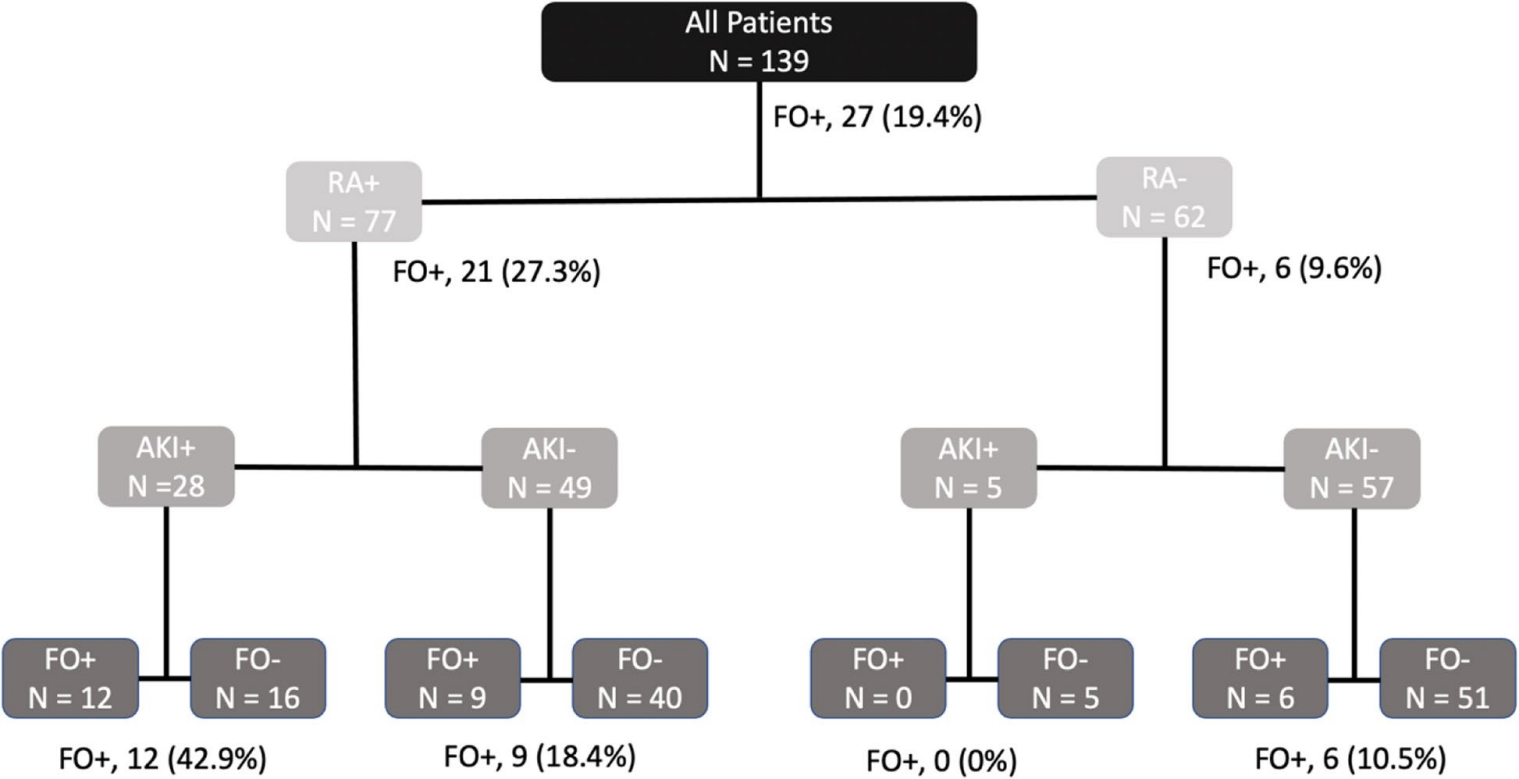
Fluid overload phenotype probability by renal angina and acute kidney injury status. RA: Renal Angina; AKI: Severe acute kidney injury; FO: Fluid overload > 15%

Four possible phenotypic states exist based on the presence or absence of FO and AKI. (Table 4). On Day 3, 15/139 (10.8%) patients had FO without AKI, 21/139 (15.1%) patients had AKI without FO, and 12/139 (8.6%) had both. RA+ conferred a negative predictive value for the phenotype development of 90.3% (80.1–96.4), 91.9% (82.2–97.3), and 100% (94.2–100%), respectively. The probability of Day 3 FO+ for RA+ patients with AKI (RA+/AKI+) was 42.9% (12/28) compared to 18.4% (9/49) for RA+/AKI-. (Figure 4) Probability of Day 3 FO+ for all RA- patients was 9.6% (6/62). No patients who were RA- developed both FO and AKI. Compared to RA-, a significantly higher proportion of RA+ developed the FO+/AKI+ phenotype (0/62 vs. 12/77, 16%, *p* = 0.001). Similarly, compared to RA+, a significantly higher proportion of RA- patients developed the FO−/AKI- phenotype (40/77, 52% vs 51/62, 82%, *p* < 0.001) (Supplemental Table 6).

**Table 4:** Fluid overloaded and acute kidney injury phenotypes and characteristics

Demographics	FO+/AKI+	FO−/AKI+	FO+/AKI-	FO−/AKI-	***P***-value
	***N*** = 12	***N*** = 21	***N*** = 15	***N*** = 91	
Male	4 (33%)	13 (62%)	7 (47%)	44 (48%)	0.45
Age (y)	3.5 (1.575–12.5)	7 (2–13)	0.75 (0.25–1.7)	6.49 (2–13)	0.003
PRISM III	14.5 (11–25.5)	9 (7–16)	4 (2–8)	5 (2–9)	< 0.001
Mechanical Vent	9 (75%)	14 (67%)	4 (27%)	46 (51%)	0.041
CRRT	9 (75%)	1 (5%)	1 (7%)	0 (0%)	< 0.001
Transplant	2 (17%)	2 (10%)	1 7%)	4 (4%)	0.39
Day 3 AKI Status					
None	0 (0%)	0 (0%)	13 (87%)	75 (82%)	< 0.001
Stage 1	0 (0%)	0 (0%)	2 (13%)	16 (18%)	0.084
Stage 2	2 (17%)	11 (52%)	0 (0%)	0 (0%)	< 0.001
Stage 3	10 (83%)	10 (48%)	0 (0%)	0 (0%)	< 0.001
Any Stage AKI	12 (100%)	21 (100%)	2 (13%)	16 (18%)	< 0.001
Severe AKI	12 (100%)	21 (100%)	0 (0%)	0 (0%)	< 0.001
Day 3 FO ≥ 15%	12 (100%)	0 (0%)	15 (100%)	0 (0%)	< 0.001
ICU LOS	14.5 (9.5–21)	6 (3–10)	4 (2–6)	6 (3–10)	0.018
Hospital LOS	32.5 (24.5–72)	16 (7–28)	14 (6–61)	11 (6–19)	0.003
Mortality	2 (17%)	2 (10%)	0 (0%)	5 (5%)	0.31
RAI Performance of Phenotype Prediction					
Sensitivity	100% (73.5–100)	76.2% (52.8–91.8)	60% (32.3–83.7)	44% (33.6–54.8)	
Specificity	48.8% (39.9–57.8)	48.3% (39–57.7)	45.2% (36.2–54.3)	22.9% (12–37.3)	
AUROC	0.74 (0.70–0.79)	0.62 (0.52–0.73)	0.53 (0.39–0.66)	0.33 (0.26–0.41)	
NPV	100% (94.2–100)	91.9% (82.2–97.3)	90.3% (80.1–96.4)	17.7% (9.2–29.5)	

Data are presented as median (IQR) for continuous measures, and n (%) for categorical measures

*RAI* Renal Angina Index, *RA-* Renal Angina Index negative, *RA+* Renal angina index positive, *PRISM III* Pediatric risk of mortality 3, *CRRT* Continuous renal replacement therapy, *AKI* Acute kidney injury, stages defined by Kidney Disease: Improving Global Outcomes (KDIGO) criteria, *FO* Fluid overload > 15%, *ICU* Intensive care unit. *LOS* Length of stay

## Discussion

In this analysis of critically ill children, renal angina fulfillment was independently associated with the development of FO at Day 3 after ICU admission. Additionally, fluid accumulation was higher among those children who fulfilled RA criteria and developed severe AKI compared to those who did not develop severe AKI. This difference was not seen among patients who did not fulfill RA criteria, which may suggest an opportunity to identify patients at highest risk of fluid accumulation.

The RAI has the ability to discriminate critically ill children who are at risk of developing severe AKI on Day 3 [9, 10]. Here, we confirm and extend these findings to fluid accumulation, with children fulfilling RAI criteria having over 5 times odds of developing FO. FO states are common among critically ill children and are associated with a large burden of resource utilization, morbidity and mortality in multiple pediatric populations [2, 15, 16, 31–34]. In particular, we find that all patients who developed the FO+/AKI+ phenotype were RA+. (Figure 4, Supplemental Table 6) Thus, RAI can identify patients at risk for severe AKI and may identify those at risk for fluid overload and therefore may benefit from fluid management strategies.

Continued investigation into the interplay of FO and AKI has demonstrated both independent and synergistic effects on the outcomes of critically ill children [6]. In our study, RAI was associated with FO status independently of severe AKI, further suggesting independent phenotypes within this population. The ability to predict the development of FO independent of AKI is important for clinicians at the bedside, offering an opportunity to intervene through directed fluid management strategies before excessive FO occurs. An ongoing prospective observational trial combining RAI with urine neutrophil gelatinase associated lipocalin (NGAL) and the furosemide stress test (FST) as a clinical support tool aims to standardize the FST and identify children at risk for FO and AKI [TAKING FOCUS 2, NCT03541785, 2P50 DK096418–06]. Trials in adult populations have demonstrated that active fluid management and deresuscitation may improve outcomes [17, 19]. With a high negative predictive value of 90% for predicting FO, RAI may provide an opportunity to identify children early in an ICU course for population enrichment for similar trials evaluating directed fluid management strategies in critically ill children.

In contrast to AKI, early FO treatment and mitigation strategies do exist and are feasible [17, 19, 20, 35–38]. While fluid administration is a mainstay of pediatric resuscitation and support, excessive fluid accumulation is common and often goes unrecognized with nearly one-third of patients with clinical signs and symptoms of FO ≥15% is unnoticed [22, 23, 39]. Major contributions to FO states tend to be from “fluid creep” as opposed to resuscitation fluids early in the ICU course and median fluid exposure exceeds weight-based maintenance on Day 3 of critical illness [22]. Here, we found that fluid accumulation was significantly different among children who developed severe AKI compared to those who did not. This finding was present as early as 36 h after ICU admission, suggesting an early opportunity to recognize and intervene before significant FO develops. Importantly, this finding was seen among patients who fulfilled RA criteria, but not among those who did not. As such, this discriminatory power of RAI may be helpful in identifying those children most at risk for excessive fluid accumulation at admission.

This study had several limitations. As a retrospective study, all findings are limited to associations as opposed to causality. Importantly, this study does not answer the cause-and-effect nature of AKI and FO. However, it does suggest the possibility to identify children early in an ICU course in a future, prospective way. Secondly, this study did not identify medications which may affect fluid balance or influence development of AKI through nephrotoxic actions. These multiple interactions should be investigated in future, prospective ways. Further, we did not find significant associations with mortality at 30 days. This finding is likely related to low overall mortality and a small cohort with inadequate power to find a difference. However, other patient- and family-centered outcomes, including dialysis receipt and hospital length of stay, were reinforced in this study. The included patients all had an expected length of stay > 48 h, which may suggest these patients were sicker and potentially bias the findings toward the development of AKI and FO. However, as the RAI was used in all patients, this inclusion factor served as a population enrichment strategy. Finally, if a patient did not have a Foley catheter in place, any mixed stool and urine output was classified as “urine output”, potentially overestimating urine output and therefore underestimating the rate of oliguria. This may bias the classification of AKI to be less severe than was actually present, potentially weakening or missing important AKI related associations.

## Conclusion

In this study of critically ill children at risk for AKI, we found that RA fulfillment was associated with increased odds of FO at Day 3. Furthermore, we demonstrated that fluid accumulation was different as early as 36 h after admission among children who went on to develop severe AKI at Day 3 compared to those who did not develop severe AKI. These findings suggest that RAI may identify patients at high risk of developing significant fluid overload.

## Supplementary Information

**Additional file 1: Supplemental Table 1.** Predictive Characteristics of RAI for FO ≥ 15%. **Supplemental Table 2.** Median FO% over time among all patients. **Supplemental Table 3.** Median FO% over time among RA+ Patients. **Supplemental Table 4.** Median FO% among RAI- patients. **Supplemental Table 5.** Predictive Characteristics of RAI for Severe AKI. Supplemental Table 6: RAI status of FO/AKI phenotypes.

## Data Availability

The data analyzed during this study are available from the corresponding author upon reasonable request.

## Abbreviations

AKI: Acute kidney injury
FO: Fluid overload
PICU: Pediatric intensive care unit
ICU: Intensive care unit
RA: Renal angina
RAI: Renal angina index
NPV: Negative predictive value
OR: Odds ratio
eGFR: Estimated glomerular filtration rate
KDIGO: Kidney diseases: improving global outcomes
IRB: Institutional review board
IQR: Interquartile range
AUROC: Area under the receiver operator characteristic curve

## References

[1] Alobaidi R, Morgan C, Basu RK, Stenson E, Featherstone R, Majumdar SR (2018). Association between fluid balance and outcomes in critically ill children: A systematic review and Meta-analysis. JAMA Pediatr.

[2] Alobaidi R, Basu RK, DeCaen A, Joffe AR, Lequier L, Pannu N (2020). Fluid accumulation in critically ill children. Crit Care Med.

[3] Alobaidi R, Morgan C, Goldstein SL, Bagshaw SM (2020). Population-based epidemiology and outcomes of acute kidney injury in critically ill children. Pediatr Crit Care Med.

[4] Sutherland SM, Ji J, Sheikhi FH, Widen E, Tian L, Alexander SR (2013). AKI in hospitalized children: epidemiology and clinical associations in a national cohort. Clin J Am Soc Nephro.

[5] Kaddourah A, Basu RK, Bagshaw SM, Goldstein SL (2017). Epidemiology of acute kidney injury in critically ill children and Young adults. New Engl J Medicine.

[6] Gist KM, Selewski DT, Brinton J, Menon S, Goldstein SL, Basu RK. Assessment of the independent and synergistic effects of fluid overload and acute kidney injury on outcomes of critically ill children. Pediatr Crit Care Med. 2019;1. 10.1097/pcc.0000000000002107. 10.1097/PCC.0000000000002107 PMC7007847 31568240

[7] Chawla LS, Bellomo R, Bihorac A, Goldstein SL, Siew ED, Bagshaw SM (2017). Acute kidney disease and renal recovery: consensus report of the acute disease quality initiative (ADQI) 16 workgroup. Nat Rev Nephrol.

[8] Basu RK, Chawla LS, Wheeler DS, Goldstein SL (2012). Renal angina: an emerging paradigm to identify children at risk for acute kidney injury. Pediatr Nephrol.

[9] Basu RK, Zappitelli M, Brunner L, Wang Y, Wong HR, Chawla LS (2014). Derivation and validation of the renal angina index to improve the prediction of acute kidney injury in critically ill children. Kidney Int.

[10] Basu RK, Kaddourah A, Goldstein SL, investigators A study, Akcan-Arikan A, Arnold M, et al. (2018). Assessment of a renal angina index for prediction of severe acute kidney injury in critically ill children: a multicentre, multinational, prospective observational study. Lancet Child Adolesc Heal.

[11] Huang L, Shi T, Quan W, Li W, Zhang L, Liu X (2020). Assessment of early renal angina index for prediction of subsequent severe acute kidney injury during septic shock in children. BMC Nephrol.

[12] Ortiz-Soriano V, Kabir S, Granado RC-D, Stromberg A, Toto RD, Moe OW, et al. Assessment of a modified renal angina index for AKI prediction in critically ill adults. Nephrol Dial Transpl. 2021. 10.1093/ndt/gfab049. 10.1093/ndt/gfab049 PMC9272422 33605426

[13] Lima L, Menon S, Goldstein SL, Basu RK (2020). Timing of fluid overload and association with patient outcome. Pediatr Crit Care Med.

[14] Akcan-Arikan A, Gebhard DJ, Arnold MA, Loftis LL, Kennedy CE (2017). Fluid overload and kidney injury score. Pediatr Crit Care Med.

[15] Sinitsky L, Walls D, Nadel S, Inwald DP (2015). Fluid overload at 48 hours is associated with respiratory morbidity but not mortality in a general PICU. Pediatr Crit Care Med.

[16] Li Y, Wang J, Bai Z, Chen J, Wang X, Pan J (2016). Early fluid overload is associated with acute kidney injury and PICU mortality in critically ill children. Eur J Pediatr.

[17] Wiedemann HP, Wheeler AP, Bernard GR, Thompson BT, Hayden D, Network NH Lung, and Blood Institute Acute Respiratory Distress Syndrome (ARDS) Clinical Trials (2006). Comparison of two fluid-management strategies in acute lung injury. New Engl J Medicine.

[18] Valentine SL, Sapru A, Higgerson RA, Spinella PC, Flori HR, Graham DA (2012). Fluid balance in critically ill children with acute lung injury. Crit Care Med.

[19] Silversides JA, Fitzgerald E, Manickavasagam US, Lapinsky SE, Nisenbaum R, Hemmings N (2018). Deresuscitation of patients with iatrogenic fluid overload is associated with reduced mortality in critical illness. Crit Care Med.

[20] Silversides JA, McAuley DF, Blackwood B, Fan E, Ferguson AJ, Marshall JC (2020). Fluid management and deresuscitation practices: A survey of critical care physicians. J Intensive Care Soc.

[21] Hassinger AB, Valentine SL (2018). Self-reported management of IV fluids and fluid accumulation in children with acute respiratory failure. Pediatr Crit Care Med.

[22] Al-Lawati ZH, Sur M, Kennedy CE, Arikan AA (2020). Profile of fluid exposure and recognition of fluid overload in critically ill children. Pediatr Crit Care Medd.

[23] Barhight MF, Nelson D, Chong G, Basu RK, Sanchez-Pinto LN. Non-resuscitation fluid in excess of hydration requirements is associated with higher mortality in critically ill children. Pediatr Res. 2021:1–6. 10.1038/s41390-021-01456-z. 10.1038/s41390-021-01456-z PMC7968408 33731814

[24] Carlton EF, Close J, Paice K, Dews A, Gorga SM, Sturza J (2020). Clinician accuracy in identifying and predicting organ dysfunction in critically ill children. Crit Care Med.

[25] Gorga SM, Carlton EF, Kohne JG, Barbaro RP, Basu RK. Consensus acute kidney injury criteria integration identifies children at risk for long-term kidney dysfunction after multiple organ dysfunction syndrome. Pediatr Nephrol. 2021:1–10. 10.1007/s00467-020-04865-0. 10.1007/s00467-020-04865-0 PMC8087651 33427986

[26] Acute Kidney Injury Work Group (2012). Kidney disease: improving global outcomes (KDIGO) KDIGO clinical practice guideline for acute kidney injury. Kidney Int Suppl.

[27] Alkandari O, Eddington KA, Hyder A, Gauvin F, Ducruet T, Gottesman R (2011). Acute kidney injury is an independent risk factor for pediatric intensive care unit mortality, longer length of stay and prolonged mechanical ventilation in critically ill children: a two-center retrospective cohort study. Crit Care.

[28] Selewski DT, Cornell TT, Heung M, Troost JP, Ehrmann BJ, Lombel RM (2014). Validation of the KDIGO acute kidney injury criteria in a pediatric critical care population. Intens Care Med.

[29] Hessey E, Ali R, Dorais M, Morissette G, Pizzi M, Rink N (2017). Evaluation of height-dependent and height-independent methods of estimating baseline serum creatinine in critically ill children. Pediatr Nephrol.

[30] Selewski DT, Askenazi DJ, Kashani K, Basu RK, Gist KM, Harer MW, et al. Quality improvement goals for pediatric acute kidney injury: pediatric applications of the 22nd acute disease quality initiative (ADQI) conference. Pediatr Nephrol. 2021:1–14. 10.1007/s00467-020-04828-5. 10.1007/s00467-020-04828-5 33433708

[31] Alobaidi R, Lequier L (2017). Fluid overload and extracorporeal membrane oxygenation. Pediatr Crit Care Med.

[32] Bagshaw SM, Brophy PD, Cruz D, Ronco C (2008). Fluid balance as a biomarker: impact of fluid overload on outcome in critically ill patients with acute kidney injury. Crit Care.

[33] Arikan AA, Zappitelli M, Goldstein SL, Naipaul A, Jefferson LS, Loftis LL (2012). Fluid overload is associated with impaired oxygenation and morbidity in critically ill children. Pediatr Crit Care Med.

[34] Hazle MA, Gajarski RJ, Yu S, Donohue J, Blatt NB (2013). Fluid overload in infants following congenital heart surgery. Pediatr Crit Care Med.

[35] Vincent J-L (2019). Fluid management in the critically ill. Kidney Int.

[36] Ostermann M, Liu K, Kashani K. Fluid management in acute kidney injury. Chest. 2019. 10.1016/j.chest.2019.04.004. 10.1016/j.chest.2019.04.004 31002784

[37] Perner A, Prowle J, Joannidis M, Young P, Hjortrup PB, Pettilä V (2017). Fluid management in acute kidney injury. Intens Care Med.

[38] Granado RC-D, Mehta RL (2016). Fluid overload in the ICU: evaluation and management. BMC Nephrol.

[39] Carcillo JA, Davis AL, Zaritsky A (1991). Role of early fluid resuscitation in pediatric septic shock. JAMA.

