# Supplementary material for: Renal angina index predicts fluid overload in critically ill children: an observational cohort study
Source: BMC Nephrol. 2021 Oct 11;22:336. doi: 10.1186/s12882-021-02540-6 (PMC8502791; doi:10.1186/s12882-021-02540-6)
Supplement: Supplementary file 1 — Additional file 1: Supplemental Table 1. Predictive Characteristics of RAI for FO ≥ 15%. Supplemental Table 2. Median FO% over time among all patients. Supplemental Table 3. Median FO% over time among RA+ Patients. Supplemental Table 4. Median FO% among RAI- patients. Supplemental Table 5. Predictive Characteristics of RAI for Severe AKI. Supplemental Table 6: RAI status of FO/AKI phenotypes. [file 12882_2021_2540_MOESM1_ESM.docx]

Supplemental Table 1: Predictive Characteristics of RAI for FO≥15%

|  | Value | 95% Confidence Interval | |
| --- | --- | --- | --- |
| Prevalence (PrA) | 19% | 13% | 17% |
| Sensitivity Pr(+\|A) | 77.8% | 57.7% | 91.4% |
| Specificity Pr(-\|N) | 50% | 40.4% | 59.6% |
| ROC area (Sens+Spec)/2 | 0.639 | 0.546 | 0.731 |
| LR+ Pr(+\|A)/ Pr(+\|N) | 1.56 | 1.18 | 2.05 |
| LR- Pr(-\|A)/ Pr(-\|N) | 0.444 | 0.214 | 0.922 |
| Odds Ratio LR(+)/LR(-) | 3.5 | 1.34 | 9.06 |
| Pos Pred Value Pr(A\|+) | 27.3% | 17.7% | 38.6% |
| Neg Pred Value Pr(N\|-) | 90.3% | 80.1% | 96.4% |

Supplemental Table 2: Median FO% over time among all patients.

Data are presented as median (IQR).

| **Hour** | **No Severe AKI** | **Severe AKI** | **p-value** |
| --- | --- | --- | --- |
|  | **N=106** | **N=33** |  |
| 24 | 1.184948 (-.1118692-2.761065) | 1.795144 (-.0330603-3.767964) | 0.26 |
| 36 | 2.182259 (-.0746682-4.297522) | 3.524592 (1.470724-7.375293) | 0.037 |
| 48 | 2.577765 (.0387932-5.034285) | 5.703953 (2.297492-9.402143) | 0.003 |
| 60 | 2.614165 (-.3069741-6.44952) | 6.464907 (1.856539-12.45859) | 0.003 |
| 72 | 3.105073 (-.1646963-7.003151) | 7.90314 (2.486962-18.60116) | 0.002 |
| 84 | 2.820181 (-1.528851-7.996439) | 8.662769 (2.494028-19.30856) | 0.001 |
| 96 | 3.131456 (-1.145315-7.876202) | 7.927011 (2.706896-22.63311) | 0.002 |

Supplemental Table 3: Median FO% over time among RA+ Patients. Data are presented as median (IQR).

| **Hour** | **No Severe AKI** | **Severe AKI** | **p-value** |
| --- | --- | --- | --- |
|  | **N=49** | **N=28** |  |
| 24 | 1.455159 (-.2125612-2.987011) | 2.405227 (.8430936-3.897485) | 0.15 |
| 36 | 2.251439 (-.0746682-4.443158) | 4.466942 (2.439597-7.513351) | 0.022 |
| 48 | 2.926065 (-.7267075-5.034285) | 7.161508 (3.005748-11.00581) | 0.002 |
| 60 | 2.944195 (-.8469664-6.712021) | 9.142323 (3.467576-15.01564) | 0.002 |
| 72 | 2.44239 (-1.619915-7.093936) | 9.754182 (3.813458-19.68939) | <0.001 |
| 84 | 1.574936 (-2.022725-8.707566) | 9.013691 (2.591404-21.70834) | <0.001 |
| 96 | .7320548 (-2.240981-11.06288) | 8.825117 (2.651295-25.38294) | 0.002 |

Supplemental Table 4: Median FO% among RAI- patients. Data are presented as median (IQR).

| **Hour** | **No Severe AKI** | **Severe AKI** | **p-value** |
| --- | --- | --- | --- |
|  | **N=57** | **N=5** |  |
| 24 | .9461168 (.0188681-2.477134) | -.1369944 (-.3614371--.0330603) | 0.054 |
| 36 | 2.07352 (-.002229-4.033145) | -1.452277 (-2.924406-2.862594) | 0.16 |
| 48 | 2.271353 (.1182855-4.896208) | 1.527876 (-3.550586-4.571563) | 0.48 |
| 60 | 2.530044 (.6949702-6.034705) | 1.856539 (-2.332993-6.207028) | 0.53 |
| 72 | 3.518759 (1.135677-6.488229) | 2.486962 (.1975645-7.90314) | 0.79 |
| 84 | 3.276241 (.1443136-6.884058) | 2.919698 (1.647915-8.662769) | 0.97 |
| 96 | 3.679861 (-.1500529-7.250078) | 3.707314 (2.891073-5.973015) | 0.77 |

Supplemental Table 5: Predictive Characteristics of RAI for Severe AKI

|  | Value | 95% Confidence Interval | |
| --- | --- | --- | --- |
| Prevalence Pr(A) | 24% | 17% | 31.7% |
| Sensitivity Pr(+\|A) | 84.8% | 68.1% | 94.9% |
| Specificity Pr(-\|N) | 53.8% | 43.8% | 63.5% |
| ROC area (Sens+Spec)/2 | 0.693 | 0.615 | 0.771 |
| LR+ Pr(+\|A)/ Pr(+\|N) | 1.84 | 1.43 | 2.36 |
| LR- Pr(-\|A)/ Pr(-\|N) | 0.282 | 0.123 | 0.644 |
| Odds Ratio LR(+)/LR(-) | 6.51 | 2.4 | 17.5 |
| Pos Pred Value Pr(A\|+) | 36.4% | 25.7% | 48.1% |
| Neg Pred Value Pr(N\|-) | 91.9% | 82.2% | 97.3% |

Supplemental Table 6: RAI status of FO/AKI phenotypes.

Data are presented as n (%).

| **Characteristic** |  | **rai = 0** | **rai = 1** | **p-value** |
| --- | --- | --- | --- | --- |
|  |  | **N=62** | **N=77** |  |
| FO+/AKI+ | 1 | 0 ( 0%) | 12 (16%) | 0.001 |
| FO-/AKI+ | 1 | 5 ( 8%) | 16 (21%) | 0.037 |
| FO+/AKI- | 1 | 6 (10%) | 9 (12%) | 0.70 |
| FO-/AKI- | 1 | 51 (82%) | 40 (52%) | <0.001 |
